# ID 353 - Cuidado Multiprofissional e a Elaboração de Projeto Terapêutico Singular no Âmbito da Atenção Primária à Saúde no Brasil, 2014-2023

**DOI:** 10.5327/2237-9622.2025.v34s1.353

**Published:** 2025-11-25

**Authors:** Thaliane Barbosa de Oliveira, Thaís Barbosa de Oliveira, Caroliny Victoria dos Santos Silva, Rafaela de Paula Sales, Aline Gonçalves Pereira

**Keywords:** Atenção Primária à Saúde, equipe Multiprofissional, tecnologia aplicada à assistência à saúde

## Abstract

**Introdução:**

O cuidado multiprofissional na Atenção Primária à Saúde (APS) é caracterizado pela aplicação de tecnologias interdisciplinares e humanizadas. Entre essas tecnologias, destaca-se o Projeto Terapêutico Singular (PTS), que consiste na elaboração de planos de cuidado personalizados, fundamentados no contexto biopsicossocial dos usuários dos serviços. A APS é estruturada por equipes que se dedicam à elaboração desses planos, com destaque às equipes Multiprofissionais (eMulti). Tais equipes se configuram como a retomada do Ministério da Saúde na atenção ao cuidado integral e multidisciplinar na APS em 2023, bem como representam a continuidade ao trabalho das equipes do Núcleo Ampliado de Saúde da Família (Nasf), cujo cofinanciamento federal foi descontinuado em 2019. Nesse contexto, este estudo teve como objetivo descrever, em uma série histórica de dez anos, as reuniões realizadas para a elaboração de PTS pelas equipes do Nasf e eMulti.

**Método:**

Realizou-se um estudo quantitativo descritivo, com dados secundários, sobre a realização de reuniões para a elaboração de PTS pelas referidas equipes, entre os anos de 2014 e 2023 no Brasil. Os dados foram extraídos da plataforma pública e on-line de Atividades Coletivas do Sistema de Informação em Saúde para a Atenção Básica (Sisab).

**Resultados:**

De 2014 a 2023, foram realizadas 423.793 reuniões para fins de elaboração de PTS pelo Nasf e eMulti na APS. O ano de 2019 destacou-se pelo percentual mais expressivo de reuniões (18,84%, n=79.862) pelo Nasf, quando comparado aos outros anos do período considerado, inclusive 2023, ano em que ocorreu a implementação das eMulti. Entre 2019 e 2023, houve um declínio de 50,19% na realização dessa atividade. Ao estratificar os dados por Grande Região, verificou-se predominância de reuniões no Sudeste (44,08%, n=186.803), seguido pelo Nordeste (29,38%, n=124.505). As Grandes Regiões Sul (14,97%, n=63.463), Centro-Oeste (6,48%, n=27.466) e Norte (5,09%, n=21.556) apresentaram menores percentuais de registros. Ademais, todas as regiões do País evidenciaram declínio na realização de reuniões para elaboração de PTS a partir de 2019.

**Conclusão:**

O maior número de reuniões destinadas à elaboração de PTS pelas equipes do Nasf no ano de 2019, seguido por um declínio nos anos subsequentes, pode ser atribuído à descontinuidade do cofinanciamento federal dessa equipe, bem como à emergência da pandemia de covid-19 em 2020. As Grandes Regiões Sudeste e Nordeste concentraram a maior quantidade de registros da atividade: a primeira destaca-se pelo seu contingente demográfico e pela implementação de equipes, enquanto a segunda detém um protagonismo histórico na implementação do Nasf em seus municípios. Em contraste, a Região Norte destaca-se pela menor frequência de reuniões voltadas à elaboração de PTS, o que pode ser explicado pelos desafios de mobilidade locorregional, de fixação de profissionais e por dificuldades no provimento de recursos humanos. Entre as limitações deste estudo, ressalta-se a impossibilidade de levantamento das categorias profissionais e dos atores intersetoriais que participaram das reuniões. Além disso, no Sisab não é possível diferenciar as produções do Nasf e da eMulti a partir de 2023, pois, para o sistema, ambas são consideradas como uma mesma modalidade de equipe. Apesar disso, os resultados indicam a necessidade de monitoramento dessas atividades para assegurar a continuidade da incorporação de PTS nos processos de trabalho multiprofissionais na APS.

